# The Role of Social Media in the Experiences of COVID-19 Among Long-Hauler Women: Qualitative Study

**DOI:** 10.2196/50443

**Published:** 2024-04-23

**Authors:** Camryn Garrett, Atefeh Aghaei, Abhishek Aggarwal, Shan Qiao

**Affiliations:** 1 Department of Health Promotion, Education, and Behavior Arnold School of Public Health University of South Carolina Columbia, SC United States

**Keywords:** COVID-19, long COVID, long-haulers, women, gender, social media, digital media, qualitative study

## Abstract

**Background:**

The extant literature suggests that women are more vulnerable to COVID-19 infection and at higher risk for developing long COVID. Due to pandemic mitigation recommendations, social media was relied upon for various aspects of daily life, likely with differences of usage between genders.

**Objective:**

This study aimed to explore the role and functions of social media in the lives of long-hauler women.

**Methods:**

Participants were purposively snowball-sampled from an online health promotion intervention for long-hauler women with COVID-19 from March to June 2021. During this time, one-on-one, semistructured interviews were conducted online until data saturation was agreed to have been achieved (ie, 15 interviews). Interview transcripts and field notes were analyzed using an emergent, inductive approach.

**Results:**

In total, 15 women were enrolled. The main roles of social media included facilitating support group participation, experience sharing, interpersonal connections, and media consumption. Emergent themes demonstrated that participants rely on social media to fulfill needs of emotional support, social engagement, spirituality, health planning, information gathering, professional support, and recreationally for relaxation. As long-hauler women turn to social media to discuss symptom and health management as well as the intention to vaccinate, this study demonstrates both the associated benefits (ie, decreased isolation) and challenges (ie, misinformation, rumination, resentment, jealousy).

**Conclusions:**

The public health implications of these findings support the development of gender-tailored health promotion interventions that leverage the benefits of social media, while mitigating the negative impacts, for women with long COVID.

## Introduction

### The COVID-19 Context

The COVID-19 pandemic, caused by SARS-CoV-2, was declared on March 11, 2020, and was accompanied by recommendations to implement prevention measures, such as masking, vaccination, testing, social distancing, isolation, and quarantine [1-5]. In the United States, as of June 16, 2023, there had been more than 103.4 million cases of COVID-19, of which more than 6.1 million required hospitalization and more than 1.1 million resulted in death [6,7]. Of those who become infected, about 30% develop postacute sequela SARS-CoV-2 (PASC), also known as chronic COVID-19 or long COVID, characterized by symptoms of varying severity that persist for 4 weeks or more after infection (eg, chronic fatigue, pain, cognitive dysfunction, muscle deconditioning, impaired concentration, and persistent ageusia and anosmia); these patients are commonly referred to as long-haulers [8-13]. Overall, women have been found to be more likely than men to develop long COVID (ie, 9.4% vs 5.5%) [14-16]. This disproportionate trend requires further investigation into the differential experiences of long COVID among women.

### Long COVID Among Women

The literature shows that there may be an association between biological sex and COVID-19 infection and recovery; however, this fails to consider the role of gender, the social environment, and gendered social norms [17]. For instance, women primarily constitute social assistance and health care workforces and face increased expectations of caregiving in the family setting, increasing the risk of COVID-19 infection [18]. As long COVID results from COVID-19 infection, differential exposure and incidence among women predisposes them to the risk for developing persisting symptoms [19]. Persistent symptoms experienced more so by long-hauler women include fatigue, difficulty breathing, muscle pain, and cognitive dysfunction, as well as the negative psychosocial outcomes of anxiety, depression, and posttraumatic stress disorder (PTSD), particularly among those who have been hospitalized [20,21].

### Social Media and COVID-19

Due to stay-at-home orders and the prioritization of social distancing as the primary means to prevent the spread of COVID-19, in the online environment, social media emerged as a key tool to adjust to the new normal, necessitating research on its roles and functions. Overall, in the United States, 97% of Americans indicate owning a cell phone of any kind, 85% indicate owning a smartphone, and 85% of US households have a broadband internet connection [22,23]. Using these technologies, 85% of US adults indicate going online at least once a day and 31% indicate that they are online “almost constantly” [22]. Among mixed findings in the COVID-19 literature, women report higher usage of social media compared to men, with assumedly differential motivations for engagement and use of platform functions [24]. Due to the prevalence of individuals being online for work and personal use, it is necessary to evaluate the role of technology and social media within the context of the pandemic and, specifically, the experiences of long-hauler women [25].

Among long-haulers specifically, social media played a vital role in developing the long-hauler identity and encouraging clinical acknowledgement. The term “long COVID” originates from social media users’ posts online [26]. Posts sharing long COVID experiences typically include a diagnosis or test result, the symptoms experienced, the length of time symptoms have persisted, an emotional response, and information and resources [27]. The growing conversations among long-haulers on social media shifted the experience of long COVID from anecdotal, exposing an invisible disability, to clinical [26,27]. With the creation of a shared identity, long-haulers were able to identify one another and further subdivided themselves into categories accounting for their intersecting identities (eg, long-hauler, woman, and mother).

Social media has been used to mitigate the impacts of lost social connections, social distancing, and isolation [21,22]. In the literature, social media has been demonstrated as a key tool used to maintain social connections, while adhering to social distancing recommendations, limiting feelings of isolation [28]. Additionally, as loneliness has been associated with decreased use of healthful coping behaviors, social media has been found to mediate the association during periods of isolation, such as during the COVID-19 pandemic [29]. There are linkages between daily use of social media and lowered measures of social isolation, as well as inversely with infrequent use of social media and higher measures of isolation [30]. Despite the positive outcomes associated with using social media during the pandemic, downfalls remain. For example, in a study focusing on older adults, internet use reflected coping efforts but did not necessarily enhance or sufficiently improve well-being [31]. Due to the mixed effects and roles of social media, throughout the pandemic, there is a unique opportunity for researchers to investigate the role of social media in social connections, isolation, and support, as well as in perpetuating access to information or misinformation among long-hauler women [32].

Overall, the literature suggests that social media sites impact users’ ability to maintain social connections, seek social support, and access information, as well as affect isolation, social comparison, and the spread of misinformation [33,34]. According to the information systems literature, gender is associated with differential motivations to use social media sites (eg, relational uses for women vs information gathering for men) and differential perceptions of information shared online [35,36]. To the best of our knowledge, despite the gendered associations relevant to social media use in other fields, few studies have assessed the differential role of social media during the pandemic, by gender, from the public health perspective. Due to an overwhelming focus on women’s experiences as essential workers and with reproductive care during the COVID-19 pandemic, there is scant literature more broadly centering on women. The experiences of women were chosen as a focus in this work due to their disproportionate burden of long COVID and their higher rates of activity and gender-specific engagement patterns on social media sites [37]. This work therefore aimed to fill a gap in the extant literature by investigating the role of social media in the experiences of long-hauler women alone.

## Methods

### Study Design

The data used in this study were derived from an online health promotion intervention for long-hauler women with COVID-19. Participants were recruited using snowball and purposive sampling through 2 social media sites, Facebook and Slack; the participants were recruited from 16 Facebook groups and 1 Slack group, as well as 2 websites of organizations for long-hauler women. Those eligible to participate in the study met the inclusion criteria of living in the United States, being aged 18 years or older, who spoke English, and who self-identified as long-haulers due to persistent COVID-19 symptoms for 4 weeks or more after infection.

### Ethical Considerations

The University of South Carolina Institutional Review Board (Pro00109358) reviewed and approved the study protocol.

Recruitment began after group and organization administrators approved posts including the study description, a flyer, and researcher contact information. The study was then advertised in each group.

### Recruitment

The recruitment period spanned 2.5 months from March to June 2021. After screening for eligibility and receiving informed consent, a total of 15 semistructured, one-on-one interviews were conducted from April to June 2021 using the online videoconferencing software Zoom [38]. Each interview lasted between 30 and 50 minutes. Participant demographics were collected through the interview process. All interviews were audio-recorded and, upon completion, field notes were written. Each participant was compensated with a US $30 e-gift card for their time and effort spent participating in the study. Data saturation was agreed to have been reached, by the 2 researchers involved in interview coding, after 15 interviews.

### Data Collection

Data were collected on the participants’ self-reported long-hauler status, the impact of persistent COVID-19 symptoms on their lives, coping strategies, and overall experiences. In these conversations, discussions of the roles of technology and social media arose organically following the semistructured interview guide. All interviews were recorded and transcribed using the service Otter.ai [39]. All artificial intelligence–derived transcripts were reviewed and verified by members of the research team. Interviewer field notes were used as additional data.

### Data Analysis

The data were analyzed following a predominately inductive approach for the thematic analysis of the interviews, as the themes identified were derived directly from the data [40-42]. The analysis process was comprised of 6 stages beginning with data familiarization and preliminary code construction, followed by the obtaining, revising, labeling, and reporting of key themes [43]. MAXQDA software was used to analyze interview transcripts [44]. In the initial phase of the thematic analysis, 2 members of the research team independently coded the transcripts, following an open coding scheme, to identify emergent themes [45-47]. The initial development of the codebook was performed after half of the interviews (ie, 7) were coded. We discussed at length the creation of the codebook to ensure accuracy of the initial codes, themes, domains, definitions, exemplar quotes, and organization. Once the codebook was finalized, the same 2 members of the research team continued to independently code the remaining transcripts. We then engaged in a collaborative review process to confirm alignment with the final codebook and to ensure consistency in the application of codes. In comparing themes, we identified similarities, differences, and interactions between themes. We used an axial coding approach to categorize the main themes and subthemes, which then guided the selection of direct quotes to demonstrate the key findings. Peer debriefing and intercoder agreement techniques were used to ensure reliability throughout the data analysis [48,49].

## Results

### Participant Details

The study participants, in alignment with the inclusion criteria, all identified as women. The participants were primarily aged between 36 and 65 years (n=12, 80%), served as essential workers (n=9, 60%), and lived with others (n=13, 87%) in the eastern region of South Carolina (n=10, 67%). Table 1 lists the participant details.

**Table 1:** Demographic characteristics of long-hauler women (N=15).

Characteristics		Participants, n (%)^a^
**Age (years)**		
	20-35	2 (13)
	36-50	6 (40)
	51-65	6 (40)
	>65	1 (7)
**Occupation**		
	Health care provider	5 (33)
	Educator	4 (27)
	Business owner	4 (27)
	Student	1 (7)
	Retiree	1 (7)
**Living situation**		
	Living with others	13 (87)
	Living alone	2 (13)
**Regional location**		
	East	10 (67)
	Central	3 (20)
	West	2 (13)

^a^The percentages might add up to more than 100 because of rounding.

### Benefits and Challenges

Long-hauler women indicated that their most used social media features included participating in support groups, posting, commenting, connecting with others, and consuming media. They used these features of social media sites to fulfill needs such as emotional support, social engagement, spirituality, health planning, information gathering, professional support, and recreation. The different functions of social media also resulted in a variety of benefits and challenges throughout the participants’ coping with long COVID. Tables 2 and 3 present the benefits and challenges related to the themes and subthemes identified and exemplar quotes.

**Table 2:** Emergent themes and subthemes of the beneficial roles of social media identified by long-hauler women.

Themes and subthemes		Exemplar quotes
**Social connection**		
	Group membership	“So I said, ‘Well, maybe if I am part of something…it is gonna be like a, like a, like a motivation for me to go through something for me. Um, because again, we are always thinking about others, you know, like, what, why am I complaining.”
	Social support	“I may not exactly know what you’re going through, but I am here to help and here to listen because a lot of times you just want a hearing—somebody to hear you.”
	Network building	“I also was posting and doing that, which, like, kept me motivated [to continue] sharing and connecting with other people…”
	Belonging	“I probably gravitated more towards that group…and I would talk about that Facebook group a lot. Like, it felt like that was like a support group, and it felt like, you know, I am not crazy, like some other people are having it, too. And I would be active in, like, commenting on, like, you know, answering people’s questions or, like, sharing, like, a connection that I have with another person that wrote on there.”
**Religiosity and spirituality**		
	Prayer	“I join[ed] an online group for praying.”
	Fellowship	The loss of a group member highlighted a sense of duty and belongingness toward one another in the online prayer group.
	Online worship	“I will go to one of my favorite pastors on YouTube and listen.”
	Meditation	“I am in a meditation group that I go to online, and we do meditation together. And then, there is, like, headspace and calm, those apps. So, there is a wide variety of different things. Like chakras, and then there is, you know, just all different kinds of relaxation and tension. Like, you squeeze your arms and look at your feet.”
**Information gathering**		
	Long hauler–shared information	“It is kind of, like, you form your own little support groups of people that had COVID. And, you know, their symptoms vary, and you are like, ‘Oh, what did you do for this?’ Or like the hair loss. That is another thing—hair loss. My hair is still not well, or whatever. And then, you know, people debating, like, ‘Are you taking the vaccine? Are you not getting the vaccine?’ So having those little groups to talk—it is good.”
	Symptom management	“I downloaded an app on my phone, and I am monitoring, like, I am documenting all of my activities for the day every day so that I can document, like, different symptoms that I am having and, like, what is, like, a trigger.”
	Physician-shared information	In reference to streaming YouTube videos: “…the different doctors and, like, what their findings are, what their recommendations are.”
**Recreation**		
	Entertainment	“I will allow myself; it does not happen every day, but, like, just to play some mind games, you know, a game of solitaire or a game…on my phone just to give myself a break.”
	Relaxation	“To help go to sleep at night. They try to, kind of, get me to relax, or whatever. And so, I think the biggest thing for me is disconnecting from all the things that I have going on, and I just…I struggle with that.”

**Table 3:** Emergent themes and subthemes of the challenging roles of social media identified by long-hauler women.

Theme and subthemes			Exemplar quotes
**Social connection**			
	Anxiety	“They can really increase anxiety.”	
	Resentment	“…it was hard. There would be resentment, and there is resentment now with the group, too, as terrible as it sounds, even jealousy, because I will see people that will write on Facebook, like, ‘Oh, like, I had COVID in December 2020’ or ‘I had COVID in January 2021,’ and a part of me just, like, would hate it because it is…like you knew, like people were advising you not to travel. And that would be what it was, especially around the holiday season, hearing people talk about, you know, having so-and-so over from, like, California, and then they got sick afterwards. And, like, it just makes you go crazy because there is so much more now. But I am trying not to, like, think like that because I mean, I do not know everyone’s experiences, and maybe, they really did avoid it or did their best to not get it, and they got it because we are in a pandemic.”	
**Information gathering**			
	Misinformation and health literacy	“Sometimes, I can understand a lot of the stuff, but there is some things that I am not as familiar with…”	
	Oversaturation and pandemic fatigue	“…after a while, like, even that [social media use] got to be so overwhelming because, again, like, everyone is, like, posting the same thing.”	

### Social Connection

A majority of the women interviewed highlighted the role of social media in reducing social isolation by providing social connection. Social connections were found to be fostered through multiple functions of social media, such as personal networks, following networks, and group membership, as well as more broadly through engagement with other users, known or unknown. Long-hauler women emphasized the importance of social media as a tool to maintain connection with their social networks when unable to be present in person. Emergent subthemes related to the main theme of social connection included the benefits of belonging fostered through social support, group membership, and network building, as well as the challenges of anxiety and resentment.

#### Group Membership and Network Building

One participant noted the function of social media in mediating “the loss of family time and not being able to be together and doing the things we have always done as a family.” Another participant described the stress associated with physical, in-person gatherings in the time of COVID-19:

I tried to host a barbecue out in our little place at the lake, and it caused me so much anxiety. I could not even eat my birthday barbecue, could not really interact with people.

At a time when minimizing physical contact with others was recommended, the online environment was found to aid in maintaining social health. Networking, a distinct feature of social media platforms, connecting individuals with others they may or may not be geographically close to, emerged as instrumental to long-hauler women’s social connection and, further, social support. Participants shared motivations for seeking membership and experiences as members of online support groups for COVID-19 long-haulers. One participant noted:

I was looking for, you know, for common ground, for folks that were experiencing some of those same things that I was, and I was also looking to support them with what I knew about my mind, body, [and] skills…

Facebook emerged as a popular social media platform among long-hauler women due to its functionality to host support groups. Many long-hauler women reported using Facebook groups to build their social networks, while also providing social and emotional support to other long-haulers. Upon reflecting on her participation in online social support groups, a participant shared:

I probably gravitated more toward that group…and I would talk about that Facebook group a lot. Like, it felt like that was like a support group, and it felt like, you know, I am not crazy, like some other people are having it, too. And I would be active in, like, commenting on, like, you know, answering people’s questions or, like, sharing, like, a connection that I have with another person that wrote on there.

Long-hauler women demonstrated the role of online groups in expanding their social networks to include other long-haulers outside their direct networks. As a result of their group membership, the majority of the participants indicated providing and receiving emotional and instrumental social support through connections fostered by membership in online support groups.

#### Social Support

Further, participants explained the role of online groups in facilitating social support from connections because “[they] are experiencing similar things that I am experiencing, so I know that it is not just me.” One participant described her role in providing emotional social support through online social connections:

I may not exactly know what you are going through, but I am here to help and here to listen because a lot of times you just want a hearing—somebody to hear you.

Participants demonstrated the crucial role of validation and affirmation as emotional social support when received from other group members regarding their emotions, symptoms, and overall experiences. Participants indicated receiving validation and affirmation when posting, commenting, and being active within their support groups. One participant discussed the benefits of continued engagement in these groups:

I also was posting and doing that, which, like, kept me motivated [to continue] sharing and connecting with other people…

As a result of providing and receiving social support within their online networks of long-hauler women, the majority of the participants report a positive effect on their sense of belonging.

#### Belonging

We found that because social media is able to connect users, participation on the platforms and in groups aids in maintaining social health through online belongingness, while also adhering to public health recommendations (eg, social distancing, isolation, quarantine). In bolstering social connections and aiding in emotional regulation, another long-hauler explained that social media motivates her to remain strong and encourages resilience. She explained:

So I said, well, maybe if I am part of something…it is gonna be like a, like a, like a motivation for me to go through something for me. Um, because again, we are always thinking about others, you know, like, what, why am I complaining.

Another participant noted the benefit of belonging to a support group:

I joined the COVID long-haulers’ Facebook group because another new thing with my shortness of breath is I noticed if I eat a lot at one time, I am way shorter of breath, and I do not know why. So, I was, like, “Oh, I am gonna see if anybody else has had these symptoms. So, I actually, like, made a post about it. And I liked that group because it makes you realize, like, you are not alone. There is all these other people that also do not have answers and also have similar symptoms as you.

Participants described how their group membership and sense of belonging decreased their feelings of loneliness and isolation, particularly when sharing experiences and symptoms with other long-haulers.

#### Anxiety and Resentment

Despite the potential benefits of participating in online groups, there remain potential consequences of participation as well. Although the findings indicated social media aids in mitigating feelings of loneliness and creating a sense of belonging, they also indicated increasing anxiety and resentment among long-hauler women. One participant noted that “they can really increase anxiety.” As related to seeing the posts of others within their social networks and in groups, a participant said:

…it was hard. There would be resentment, and there is resentment now with the group, too, as terrible as it sounds, even jealousy, because I will see people that will write on Facebook like “Oh, like, I had COVID in December 2020” or “I had COVID in January 2021,” and a part of me just, like, would hate it because it is…like you knew, like people were advising you not to travel. And that would be what it was, especially around the holiday season, hearing people talk about, you know, having so-and-so over from, Like, California, and then they got sick afterwards. And, like, it just makes you go crazy because there is so much more now. But I am trying not to, like, think like that because I mean, I do not know everyone's experiences, and maybe, they really did avoid it or did their best to not get it, and they got it because we are in a pandemic. But it is stuff like that. Like, I feel like I am more, like, insecure with my experience. I get jealous of other people's experiences. There's just, like, a lot of negative-ness with it…

In sharing this anecdote, the participant voiced her frustration toward and resentment of those who, after participating in high-risk activities, shared their COVID-19 experiences online. Engagement with such individuals and their posts then led to this participant’s insecurity in their own experiences.

### Religiosity and Spirituality

In addition to the impacts of social media on social health, participants highlighted its role in also maintaining their spiritual health. In addition to joining online groups topically centered around COVID-19, a participant indicated, “I join[ed] an online group for praying.” She detailed the group, demonstrating its resemblance to that of other support groups, albeit not solely related to COVID-19, with the added element of religion. Overall, the participant’s sentiments indicated that the group positively impacted her overall well-being. When describing the loss of a member of the group, she highlighted the role of fellowship and connection in the group as they lifted one another up in prayer and, in doing so, created belongingness, community, and strength.

Another participant demonstrated the role of social media as related to religiosity and spirituality by noting her use of video-streaming platforms to seek spiritual support. She described her engagement as, “I'll go to one of my favorite pastors on YouTube and listen.” During a time when physically gathering with others, as in the case of congregating for religious observances, was considered high risk, social media provided an avenue through which long-haulers could maintain their spiritual practices. Relatedly, participants indicated using social media to engage in guided meditations. One shared her daughter’s role in encouraging her participation:

She gave me some resources online, in an app, and then my daughter uses a different…she uses Spotify. So, she gave me that information, and so I kind of just went off of those suggestions, and now, I have my favorite guided meditations that I use on Spotify, and they are effective.

Other participants said that they similarly engage in guided meditations but also participate in groups specific for meditation and relaxation. One participant described the meditation group and smartphone apps used:

I am in a meditation group that I go to online, and we do meditation together. And then, there is, like, headspace and calm—those apps. So, there is a wide variety of different things. Like chakras, and then there is, you know, just all different kinds of relaxation and tension. Like you squeeze your arms and look at your feet.

### Information Gathering

Apart from social networking, one of the most prominent functions of social media is the sharing of news and information. Within the context of the COVID-19 pandemic, social media served as a conduit for sharing COVID-19 news, government policies and announcements, updated prevention guidelines, and general information. The findings demonstrated that long-hauler women used social media to seek information related to COVID-19 vaccines, symptoms, and symptom management strategies, as well as to follow news related to emerging treatments.

#### Long Hauler–Shared Information

Regarding COVID-19 information, topics of interest were primarily related to symptoms and health management. Long-hauler women indicated turning to online support groups to gather information from those with similar experiences. One participant illustrated the symptom and health discussions within these groups:

It is kind of like you form your own little support groups of people that had COVID. And, you know, their symptoms vary, and you are like, “Oh, what did you do for this?” Or like the hair loss. That is another thing—hair loss. My hair is still not well, or whatever. And then, you know, people debating, like, “Are you taking the vaccine? Are you not getting the vaccine?” So having those little groups to talk—it is good.

Alongside using support groups for discussion, long-haulers indicated also using smartphone apps to track symptoms and create health care plans. One long-hauler discussed her experience:

I downloaded an app on my phone, and I am monitoring, like, I am documenting all of my activities for the day every day so that I can document, like, different symptoms that I am having and, like, what is, like, a trigger.

Due to the persistent nature of COVID-19 symptoms experienced by long-haulers, monitoring symptoms is in the interest of patients to aid in symptom management and for use with health care providers in creating treatment plans.

#### Physician-Shared Information

In addition to sharing information across networks of long-haulers on social media, participants also noted gathering information through online interactions with physicians and mental health professionals. One participant indicated obtaining pandemic-related information from physicians on YouTube as she watched “…the different doctors and, like, what their findings are, what their recommendations are.” Due to the increasing burden of mental health challenges coupled with physical symptoms, as expressed by the participants, social media offers a platform for mental health resource sharing, at a time when many cannot access needed services. One participant detailed these difficulties:

I had been looking for, like, counseling, and a lot of the counseling in our plan has, like, basically stopped taking people. Like, I think it is, like, kind of like, overwhelmed right now, and, like, I would call, like, a whole list, and I would go through the whole list, and, like, they are not taking new patients. So, I just have to be persistent about it.

In coping with barriers (ie, wait lists, cost) to accessing mental health services, participants indicated using social media as a tool to gain information from professionals. For instance, a participant said that she “join[ed] a group…they had a list of faculty members that were starting groups…you did not have to pay for it.” This participant was able to engage in mental health services through a free and accessible online group operated by mental health professionals. This function of social media is valuable in responding to increasing mental health needs by addressing barriers to accessing professional psychological support.

#### Misinformation and Health Literacy

The potential consequences of users obtaining information from social media, particularly that which must be scientifically based, include a lack of or difficulty in understanding, as well as the distribution of and access to unvalidated content or misinformation. Illustrating the difficulty in understanding sought-out information, a participant shared:

Sometimes I can understand a lot of the stuff, but there is some things that I am not as familiar with…

Due to the evolving nature of scientific discovery over the course of the pandemic, there were difficulties in grasping timelines and emergent findings that inhibited understanding and perpetuated misunderstanding. In the case of long-haulers, their increased need for health care exposes them to complex medical jargon that may require a higher level of health literacy to mitigate misunderstanding. Overall, due to the need for regularly updated COVID-19 information, social media functions as both a benefit and a hindrance to its dissemination. Social media provides users with increased access to information, while also providing a platform through which misinformation may be widely shared.

#### Oversaturation and Pandemic Fatigue

Further, despite the benefits of engaging in support groups and accessing pandemic-related information online, participants indicated differing perspectives on the amount of information shared. Referencing a long-hauler Facebook support group, a participant noted:

And, like, the nice thing about it is they share loads of information.

Alternatively, another participant shared that due to the sameness and sheer volume of pandemic-related content on social media:

…after a while, like, even that [social media use] got to be so overwhelming because, again, like everyone is, like, posting the same thing.

Due to oversaturation and misinformation, a participant noted that she is “disappointed with social media.” This disappointment has kept the participant from participating in COVID-19 and long-hauler groups.

### Recreation

In addition to the networking and information-gathering functionalities of social media, participants also indicated leveraging social media for entertainment, recreation, and relaxation. In coping with their diagnosis, symptoms, anxiety, and the state of the world, long-hauler women indicated using social media and smartphone apps to play games, watch videos, and listen to music. One participant described consuming content on social media as a method to cope with the anxiety of attending post–COVID-19 appointments. Another participant shared:

I will allow myself; it does not happen every day, but, like, just to play some mind games, you know, a game of solitaire or a game…on my phone just to give myself a break.

Another participant indicated using the social media site YouTube as a way “to help go to sleep at night.”

They try to, kind of, get me to relax or whatever. And so, I think the biggest thing for me is disconnecting from all the things that I have going on, and I just…I struggle with that.

These findings suggest that social media is a method by which participants seek entertainment, recreate, and relax. These functions serve as social media–based coping mechanisms to alleviate mental health burdens.

## Discussion

### Principal Findings

Long-hauler women identify engagement in online support groups to be a primary use of social media during the COVID-19 pandemic. These groups are typically disease specific and can be described as communities where individuals can congregate and engage in broader group discussions as a form of social connection [50]. Support groups function to allow members to affirm their long-hauler identity, maintain connections, combat isolation, seek support, compare experiences, share remedies, and coruminate [51-53]. Long-hauler women seek reassurance through channels of connection with others who share their disease-specific identity and to cope with a social environment characterized by mortality, unemployment, resource loss, and psychological burdens of prevention measure adherence and disease [54].

Online support groups have been previously assessed in various disease contexts in the literature. A systematic review of the role of online support groups for patients with prostate cancer found that the groups not only aided in participant decision-making through their dissemination and exchange of information but also provided participants with social support [55]. A review of support groups for patients with breast cancer demonstrated that the benefits or consequences of participation in social support groups are inconclusive [56]. A systematic review of studies assessing the impacts of social support groups on patients with chronic conditions found that they demonstrate a wide array of support group implementation and outcome measurements that complicate their use in the context of the COVID-19 pandemic [57]. Within the context of COVID-19, online support groups act as a tool to comply with social distancing guidance, while maintaining connections and combating isolation, depression, and anxiety [51,52]. Additionally, a systematic review of COVID-19–specific social support groups demonstrated that although they are effective in addressing participants’ psychological and psychosocial needs, due to their responsiveness to the emerging needs and challenges faced by participants, there remains a need for further research [58].

This study presented both benefits and challenges associated with the participation of long-hauler women in COVID-19–specific social support groups. The benefits include the validation of shared experiences, decreased isolation, and motivation to pursue symptom management and recovery. The challenges for long-hauler women’s participation include experiences of increased anxiety due to rumination within the groups, resentment and jealousy due to others’ posts of unsafe pandemic activities or recovery, and an insecurity of experiences as a result of comparison. Additionally, there is the complication of pandemic fatigue, as instigated by the overwhelming amount of posts within social support groups. Within the extant literature, overexposure to pandemic news may act as a disaster stressor that acts as a risk factor for negative psychosocial outcomes [59]. As demonstrated through these findings, related to social support and network building, social media presents an opportunity for individuals to receive support and engagement with others, while also facing potential, associated challenges.

Additional engagement on social media revolved around spirituality, entertainment, recreation, and relaxation. Beyond disease-specific support groups, long-hauler women reported relying on groups that specifically serve to maintain spiritual health. Digital media, more broadly, allows long-hauler women to engage in spiritual practices alone or with others, as desired. These novel functions are significant as spiritual health has been identified as a key coping mechanism to facilitate resilience [60]. Further, digital media has demonstrated its usefulness in the coping of long-hauler women, as they noted its use for entertainment, recreation, and relaxation through audio and visual content. The emerging pandemic literature has sought to assess the complex benefits and consequences of media usage, motivations, stress, and psychosocial outcomes that have been found to differ by demographics [61]. Despite the complex mechanisms of coping within the literature, long-hauler women in this study identified digital and social media used for entertainment to be a positive coping strategy.

In addition to the features of social media facilitating coping, long-hauler women also relied on networking sites to access pandemic-related information. One unique feature of social media is the unprecedented speed with which information can be shared, particularly evolving pandemic information, but it also presents the risk of misinformation and associated difficulties in mitigating its negative impacts [62,63]. A key consequence, due to the nature of social media, is the tendency for information that sparks outrage, typically containing misinformation, to move the most quickly through social channels, likely stifling needed, correct information [63]. Therefore, as outrage impacts the visibility of trending topics, depending on the content, it can alter individuals’ risk perceptions [63]. This study corroborated these trends due to participants’ disappointment with the distribution of information and with social media overall. Additionally, when evaluating what constitutes misinformation, it is necessary to consider nuances in the perspectives of various key players (eg, patients, providers, scientists), as well as their potential contributions to the knowledge base (eg, symptomology, diagnostic criteria).

As social media content allows misinformation to trend due to its outrage-evoking characteristics, the COVID-19 pandemic is seen as syndemic with an infodemic. Within the infodemic, long-hauler women expressed experiencing difficulty in understanding health information presented online as related to vaccines, symptom management, and news. In addition to the threats posed by misinformation to public health prevention efforts, social media presents users with a plethora of information that operates to mitigate the associated negative effects [33]. Social and cultural factors influencing the perceptions of and responses to health information and risk communication are related to personal control, uncertainty, trust in institutions, and trust in media, as well as an overall sense of immediacy [63].

Associated with COVID-19 information, long-hauler women turn to social media and online social support groups to discuss symptom and health management, as well as the intention to vaccinate. Digital media, beyond social media, has benefited women with chronic COVID-19, allowing them to document and track their symptoms. As chronic COVID-19 is characterized by persistent symptoms, symptom management support and remedy information sharing were found to be salient uses of social media among women with long COVID. This finding aligns with evidence within the extant literature where social media has been used throughout the pandemic, by broader populations, to share medication strategies, anonymously seek information, crowdsource information, and engage in advocacy [64-67]. Overall, social media is used by long-hauler women to cope, exchange social support, maintain spirituality, and seek entertainment, while also disseminating information relevant to the long-hauler experience.

### Strengths and Limitations

Our findings are in alignment with “uses and gratifications theory,” which posits motivations for social media use as revolving around meeting certain needs, including social connection, knowledge, and relaxation, among others [68,69]. This study contributes to the sparce, evolving literature, with findings focusing on the social media usage of long-hauler women specifically.

Our study is also subject to several limitations. First, there were constraints on the analysis due to a small sample size with limited demographic variability. Second, the structure of the questions asked restricted our ability to identify patterns of usage by platform, relying, rather, on broader trends. Additionally, as support groups were used for recruitment, the findings may not be representative of the experiences of women not engaged on social media or in online support groups.

### Public Health Implications

Although this study is additive to the evolving literature, strengthening the present evidence base beyond quantitative, descriptive analyses that do not account for gendered experiences, it demonstrates a need for further research. Future deductive work should consider, concurrently, comparing the uses of social media across the spectrum of gender and age based on known differences in usage. Due to the reliance on social media platforms to gather knowledge, further work is necessitated to evaluate the content and quality of information shared within online discussions and support groups. Future research should use an intersectional framework to assess the role of social media across a variety of additional identities women hold (eg, race/ ethnicity, preexisting conditions, socioeconomic status).

### Conclusion

The findings of this study support the development of gender-tailored health promotion interventions that leverage the benefits of social media, while mitigating the consequences, for women with chronic COVID. As social media serves as a pandemic mitigation tool, there is a need to better understand patterns and experiences of usage [70-72]. Informed by our findings, long-hauler women should be met where they are, through the platforms and functions that they currently use, in order for public health interventions to aid them in managing long COVID and its associated effects.
